# Antimicrobial resistance in paediatric bloodstream infections in Tanzania: a longitudinal comparison of two cohort studies

**DOI:** 10.1186/s12866-026-05024-5

**Published:** 2026-04-14

**Authors:** Trygve Kristiansen, Sabrina John Moyo, Joel Manyahi, Paul Christoffer Lindemann, Iain G. Johnston, Kristine Mørch, Nina Langeland, Bjørn Blomberg

**Affiliations:** 1 Department of Clinical Science, University of Bergen, Jonas Lies Veg 65, 5021 Bergen, Norway; 2 National Centre for Tropical Infectious Diseases, Department of Medicine, Haukeland University Hospital, Bergen, Norway; 3 Department of Tropical Disease Biology, Liverpool School of Tropical Medicine, Liverpool, UK; 4 Department of Microbiology and Immunology, Muhimbili University of Health and Allied Sciences, Dar Es Salaam, Tanzania; 5 Department of Medical Microbiology, Haukeland University Hospital, Bergen, Norway; 6 Department of Mathematics, University of Bergen, Bergen, Norway; 7 Computational Biology Unit, University of Bergen, Bergen, Norway; 8 Norwegian Institute of Public Health, Oslo, Norway

**Keywords:** Paediatric sepsis, Bloodstream infection, Bacterial drug resistance, Aminoglycosides, United Republic of Tanzania

## Abstract

**Background:**

Paediatric sepsis remains a significant global health issue, with the highest burden in low- and middle-income countries. Widespread antimicrobial resistance makes standard first-line empiric treatment regimens ineffective. To explore potential alternatives, we compared antimicrobial resistance patterns in blood culture isolates from two previous cohort studies on children admitted to hospital with fever in Tanzania, with focus on resistance to the semisynthetic aminoglycosides amikacin and plazomicin.

**Methods:**

Antimicrobial susceptibility testing was conducted using minimum inhibitory concentration (MIC) strips, with results interpreted according to European Committee on Antimicrobial Susceptibility Testing (EUCAST) clinical breakpoints. Resistance rates to current recommended empiric sepsis treatments and potential new regimens with ampicillin combined with amikacin or plazomicin were assessed. Whole genome sequencing was performed on *Klebsiella*, *Escherichia*, and *Salmonella* isolates.

**Results:**

Antimicrobial susceptibility testing was conducted on 449 blood culture isolates from 427 patients, and whole genome sequencing was performed on 216 isolates. Overall resistance rates to gentamicin-ampicillin and ceftriaxone were 46% and 50%, respectively, while resistance to amikacin-ampicillin and plazomicin-ampicillin was 9%. We estimate that by using amikacin-ampicillin instead of gentamicin-ampicillin, we can reduce the risk of ineffective antibiotic treatment by 37 percentage points (95% confidence interval: 32%-42%). Correspondingly, using amikacin-ampicillin instead of ceftriaxone would reduce the risk by 41 percentage points (95% confidence interval: 35%-46%). In *Klebsiella pneumoniae* and *Escherichia coli* ceftriaxone resistance primarily resulted from the *bla*_CTX-M-15_ gene, while resistance to gentamicin was mainly due to the aac(3)-II gene. The aac(6′)-Ib-cr gene was found in 33 *Klebsiella* and *E. coli* isolates, although only three exhibited amikacin MICs above the clinical breakpoint.

**Conclusion:**

This in vitro analysis suggest that amikacin-ampicillin is a promising option as first-line empiric treatment of suspected sepsis in children in Tanzania. The clinical efficacy and safety need to be evaluated in clinical trials.

**Supplementary Information:**

The online version contains supplementary material available at 10.1186/s12866-026-05024-5.

## Introduction

Sepsis remains a leading contributor to global morbidity and mortality, disproportionately affecting children in low- and middle-income countries (LMICs) across sub-Saharan Africa [1–6]. Main challenges in managing sepsis in LMICs are inadequate diagnosis leading to non-targeted antimicrobial treatment, and widespread antimicrobial resistance (AMR) that renders standard treatment regimens ineffective. The African region is estimated to bear the highest burden of AMR, with 250 000 deaths directly caused by antibiotic-resistant bacteria annually and children, particularly neonates, are especially vulnerable [7–10].

Paediatric sepsis is defined as suspected infection in a child who additionally has life-threatening organ dysfunction, indicated by a Phoenix Sepsis Score of ≥ 2 [11]. Although malaria, viral infections, and non-culturable bacteria account for a substantial proportion of sepsis cases, blood culture findings remain the cornerstone for defining the aetiology of bacterial sepsis across populations and serve as a key source of data for epidemiological studies and global burden estimates [2–4, 6]. The most common bacterial pathogens causing sepsis in children in LMICs in sub-Saharan Africa are *Staphylococcus aureus*, *Klebsiella pneumoniae* and *Escherichia coli* [2, 5, 12–14]. Antibiotic treatment is initiated based on the clinical suspicion of sepsis, and in many sub-Saharan countries, the most common empiric treatment for suspected sepsis in children is either a penicillin combined with gentamicin or ceftriaxone monotherapy [15–17]. However, this established treatment is ineffective in a high proportion of patients, mainly due to the high prevalence of extended-spectrum beta-lactamase (ESBL) production and gentamicin resistance in Gram-negative bacilli and methicillin-resistant *S. aureus.* [13, 15, 18]. Therefore, there is an urgent need for alternative empiric treatment regimens to improve clinical outcome and possibly counteract the growing AMR problem.

Improved understanding of antimicrobial resistance patterns in clinical isolates may inform new treatment options. Empirical treatment with a penicillin combined with an aminoglycoside offers potential advantages as it causes less collateral damage to the gut microbiota and has a lower potential for inducing AMR compared to broad-spectrum β-lactams [19–23]. Semisynthetic aminoglycosides are developed to evade aminoglycoside-modifying enzymes that inactivate gentamicin [24–26]. In this study, we analyse the in vitro antibiotic resistance in blood culture isolates from two prior cohort studies on children admitted to hospital with fever in Dar es Salaam, Tanzania [27, 28], to evaluate the resistance to potential new treatment regimens with the semisynthetic aminoglycosides amikacin and plazomicin compared with established empiric treatment.

## Methods

### Study design and participants

Blood culture isolates were collected in two previous prospective cohort studies of children consecutively admitted due to febrile illness or suspected severe infections at hospitals in Dar es Salaam, Tanzania. Participants were only included in the study if the parent or responsible caretaker provided informed, written consent. Study 1 took place at Muhimbili National Hospital from August 2001 to August 2002 and included children aged 0–7 years old [27]. Study 2 took place at Muhimbili National Hospital and 3 regional hospitals, Amana, Temeke and Mwananyamala, from March 2017 to July 2018 and included children aged 0–5 years old [28]. The sample sizes were decided pragmatically including all patients available during the study periods, *n* = 1828 for Study 1 and *n* = 2226 for Study 2. A single blood culture was obtained from each patient. Coagulase-negative staphylococci and bacteria of doubtful pathogenicity were excluded. Anaerobic culture was not performed.

### Antimicrobial susceptibility testing

For antimicrobial susceptibility analysis, minimum inhibitory concentrations (MICs) were determined using Liofilchem MIC Test Strips (Liofilchem, Italy) according to the manufacturer's instructions. Results were interpreted using the European Committee on Antimicrobial Susceptibility Testing (EUCAST) clinical breakpoints (version 14.0). As the EUCAST guidelines currently does not have breakpoints for plazomicin, results for this antibiotic were interpreted using the Clinical and Laboratory Standards Institute (CLSI) clinical breakpoints (M100 ED34:2024) and MIC distributions from published studies [29–31]. When no breakpoints were available, MIC distribution and epidemiological cut-off values were used to distinguish isolates with acquired resistance mechanisms from those without (wild type). Ceftriaxone resistance in *Staphylococcus aureus* was interpreted from the cefoxitin disc diffusion test and confirmed with mecA PCR. *Klebsiella* spp. and AmpC-producing *Enterobacterales* were considered intrinsically resistant to ampicillin. *Pseudomonas *spp. were considered resistant to gentamicin and ceftriaxone. *Acinetobacter *spp. were considered resistant to ceftriaxone. *Enterococcus *spp. and *Streptococcus *spp. were considered intrinsically resistant to aminoglycosides. Aminoglycoside breakpoints were applied for all patients regardless of the focus of infection.

### Whole genome sequencing and analysis

Whole genome sequencing (WGS) was performed using the Next Generation Sequencing platform (Illumina, San Diego, CA, USA), HiSeq X10 (Microbes NG, Birmingham, UK), and MiSeq (Haukeland University Hospital, Bergen, Norway). WGS data were analysed using SeqSphere + software (Ridom GmbH, Münster, Germany) [32]. If the phenotypic resistance could not be explained by the predicted phenotype using NCBI AMRFinder in SeqSphere +, the resistance genes were identified from the raw reads using ResFinder (version 4.7.2) [33]. β-lactamases were classified as “narrow spectrum β-lactamase” or “extended spectrum β-lactamase (ESBL)” according to the β-lactamases database BLBD.eu [34]. *Bla*_SHV_ genes in *Klebsiella* were classified according to Tsang et al. [35].

### Statistical analyses

Resistance was assessed at the patient level and defined as in vitro resistance to a regimen if any pathogenic isolate was resistant to its antibiotics; for combination regimens, resistance required an isolate resistant to both agents. The proportion of patients with isolates resistant to different treatment regimens was calculated for each regimen and stratified by bacterial species. Separate analyses were performed for neonates and children > 28 days. To compare resistance proportions between combination regimens, we calculated the absolute difference in percentage points. The 95% confidence interval for the absolute resistance difference was estimated using the standard error for the difference between two proportions. Differences in MIC values were assessed by Wilcoxon test. All statistical analyses and visualization were conducted in R v4.5.0 [36] using the readxl [37], tidyverse [38], dplyr [39], ggplot2 [40], and forestplot [41] packages.

## Results

Of 4054 children in the two cohorts, 11% (*n* = 427) had bloodstream infections: 12% (211/1828) of participants in Study 1 and 10% (216/2226) in Study 2. Overall, 52% (223/427) of those with bloodstream infections were neonates (≤ 28 days), and 41% (175/427) were girls. The median age was 15 days (range 0–2654 days), and the mean age was 259 days. Of 521 blood culture isolates from the original studies, 51 (10%) were excluded. *Candida* spp. (*N* = 32) and *Mycobacterium tuberculosis* (*N* = 1) were excluded because these genera cannot be treated with regular antibiotics and thus were considered outside the scope of this study. Additionally, 18 isolates were excluded due to doubtful pathogenicity, seven isolates showed no growth, and 14 isolates were missing, resulting in a total of 449 blood culture isolates from 427 patients available for antimicrobial susceptibility testing. The most common Gram-negative bacteria were *K. pneumoniae* (*N* = 113), *E. coli* (*N* = 60), and *Salmonella enterica* (*N* = 46). All but two *K. pneumoniae* and one *E. coli* isolates were whole genome sequenced. *Salmonella* isolates had the following serovar distribution: Enteritidis (*N* = 20), Typhimurium (*N* = 19), Typhi (*N* = 5), Indiana (*N* = 1), and Newport (*N* = 1). The most common Gram-positive bacteria were *S. aureus* (*N* = 72) and *Enterococcus *spp. (*N* = 67). Twenty patients had more than one bacterial species in blood cultures and were considered to have polymicrobial infections.

Table 1 shows aggregated resistance rates from both studies for standard treatments and for amikacin or plazomicin in combination with ampicillin. The overall resistance rates to gentamicin-ampicillin, gentamicin-ampicillin-cloxacillin and ceftriaxone were 46%, 45% and 50%, respectively. In *Enterobacterales*, resistance rates were highest for *K. pneumoniae*, with a 66% resistance rate to gentamicin-ampicillin and 57% to ceftriaxone. Overall resistance rates to ciprofloxacin were 30%, with 16% resistance in *Klebsiella* and 24% in *E. coli*. The overall resistance rates to ampicillin combined with either amikacin or plazomicin were 9%. Resistance to these regimens was primarily caused by *Enterococcus faecium* resistant to ampicillin. Overall resistance rate to amikacin-ceftriaxone was 17%, primarily due to intrinsic resistance in enterococci. MIC distributions for amikacin in *Enterobacterales*, *Acinetobacter *spp*.*, *Pseudomonas *spp*.*, and *S. aureus* are shown in Supplementary Fig. 1. Supplementary Table 1 and 2 shows resistance rates in neonates and in children > 28 days of age, respectively.

**Table 1:** In vitro antimicrobial resistance rates to different potential empiric sepsis treatment regimens in 449 blood culture isolates from 427 children with bloodstream infections in Tanzania

	Gentamicin + Ampicillin	Gentamicin + Ampicillin + Cloxacillin	Ceftriaxone	Ciprofloxacin	Amikacin + Ampicillin	Plazomicin + Ampicillin	Amikacin + Ceftriaxone
Gram-negative bacteria							
*Klebsiella pneumoniae* (*N* = 100)	66% (*n* = 66)	66% (*n* = 66)	57% (*n* = 57)	16% (*n* = 16)	0% (*n* = 0)	0% (*n* = 0)	0% (*n* = 0)
*Escherichia coli* (*N* = 55)	33% (*n* = 18)	33% (*n* = 18)	29% (*n* = 16)	24% (*n* = 13)	4% (*n* = 2)	0% (*n* = 0)	4% (*n* = 2)
*Salmonella enterica* (*N* = 42)	31% (*n* = 13)	31% (*n* = 13)	2% (*n* = 1)	7% (*n* = 3)	0% (*n* = 0)	0% (*n* = 0)	0% (*n* = 0)
Other Gram negatives^a^ (*N* = 30)	20% (*n* = 6)	20% (*n* = 6)	20% (n = 6)	7% (*n* = 2)	0% (*n* = 0)	3% (*n* = 1)	0% (*n* = 0)
* Pseudomonas *spp*. * (*N* = 22)	100% (*n* = 22)	100% (*n* = 22)	100% (*n* = 22)	5% (*n* = 1)	0% (*n* = 0)	0% (*n* = 0)	0% (*n* = 0)
* Acinetobacter *spp*.* (*N* = 22)	50% (*n* = 11)	50% (*n* = 11)	100% (*n* = 22)	18% (*n* = 4)	14% (*n* = 3)	9% (*n* = 2)	14% (*n* = 3)
Gram-positive bacteria							
*Staphylococcus aureus* (*N* = 67)	24% (*n* = 16)	15% (*n* = 10)	19% (*n* = 13)	15% (*n* = 10)	0% (*n* = 0)	0% (*n* = 0)	0% (*n* = 0)
*Enterococcus *spp*.* (*N* = 63)	51% (*n* = 32)	51% (*n* = 32)	100% (*n* = 63)	100% (*n* = 63)	51% (*n* = 32)	51% (*n* = 32)	100% (*n* = 63)
*Streptococcus *spp*.* (*N* = 6)	0% (*n* = 0)	0% (*n* = 0)	0% (*n* = 0)	100% (*n* = 6)	0% (*n* = 0)	0% (*n* = 0)	0% (*n* = 0)
Polymicrobial infections							
Polymicrobial^b^ (*N* = 20)	70% (*n* = 14)	70% (*n* = 14)	70% (*n* = 14)	40% (*n* = 8)	15% (*n* = 3)	15% (*n* = 3)	30% (*n* = 6)
Overall (*N* = 427)	46% (*n* = 198)	45% (*n* = 192)	50% (*n* = 214)	30% (*n* = 126)	9% (*n* = 40)	9% (*n* = 38)	17% (*n* = 74)

^a^Other Gram-negatives: *Enterobacterales* (*N* = 29), *Elizabethkingia anophelis* (*N* = 1)

^b^Polymicrobial: *Klebsiella* + *E. coli* (*N* = 4), *S. aureus* + *Enterococcus* (*N* = 2), *Klebsiella* + *Pantoea* (*N* = 1), *Klebsiella* + *Proteus* (*N* = 1), *Klebsiella* + *Enterobacter* (*N* = 1), *Klebsiella *+ *Citrobacter* + *Enterobacter* (*N* = 1),  *E. coli* + *Pseudomonas* (*N* = 1), *Klebsiella* + *Acinetobacter* (*N* = 1), *Klebsiella* + *Enterococcus* (*N* = 1),  *Klebsiella* + *Pseudomonas* (*N* = 1), *Klebsiella* + *Salmonella* (*N* = 1), *Klebsiella* + *Salmonella*  +  *Enterococcus* (*N* = 1), *S. aureus* + *Serratia* (*N* = 1), *S. aureus* + *Streptococcus agalactiae* (*N* = 1), *Salmonella *+ *Pseudomonas *(*N* = 1), *Salmonella* + *S. aureus* (*N* = 1)

**Fig. 1:**
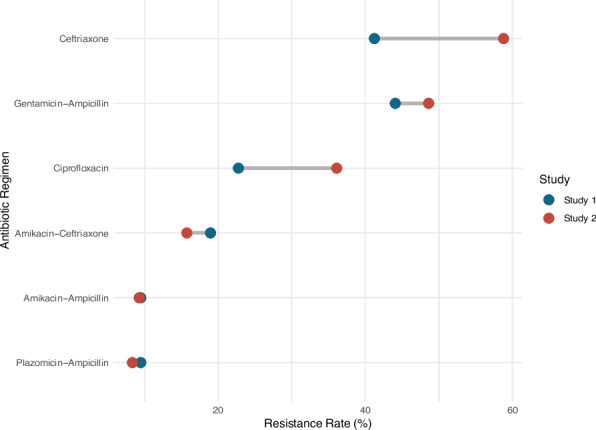
Change in overall resistance rates to different potential sepsis treatment regimens between Study 1 (2001–2002) and Study 2 (2017–2018)

Between Study 1 (2001–2002) and Study 2 (2017–2018), overall resistance rates to gentamicin-ampicillin increased from 44 to 49%, resistance to ceftriaxone from 41 to 59%, and resistance to ciprofloxacin from 23 to 36% (Fig. 1). Resistance to ampicillin combined with amikacin or plazomicin was similar in the two studies. Resistance rates in Study 1 and Study 2 are summarized in Supplementary Tables 3 and 4.

**Fig. 2:**
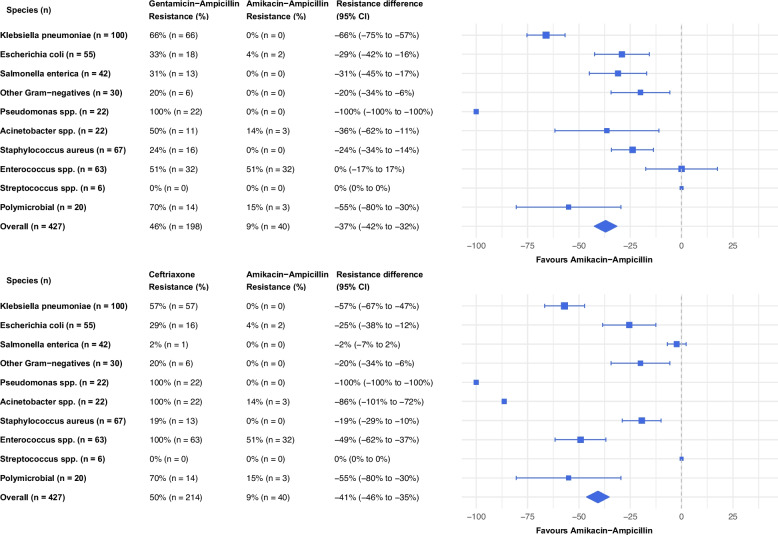
Absolute risk differences for resistance rates to amikacin-ampicillin compared to gentamicin-ampicillin and ceftriaxone across different species and overall. CI, confidence interval

The absolute risk difference in resistance rates was 37 percentage points (95% confidence interval 32%–42%) for amikacin–ampicillin versus gentamicin–ampicillin, and 41 percentage points (95% confidence interval 35%–46%) versus ceftriaxone. Risk reduction with amikacin-ampicillin was particularly notable for *K. pneumoniae*, showing a 66 and 57 percentage points reduction compared to gentamicin-ampicillin and ceftriaxone, respectively (Fig. 2). Risk differences for resistance across the different treatment regimens were similar for neonates and children > 28 days of age (Supplementary Fig. 2 a-d).

**Fig. 3:**
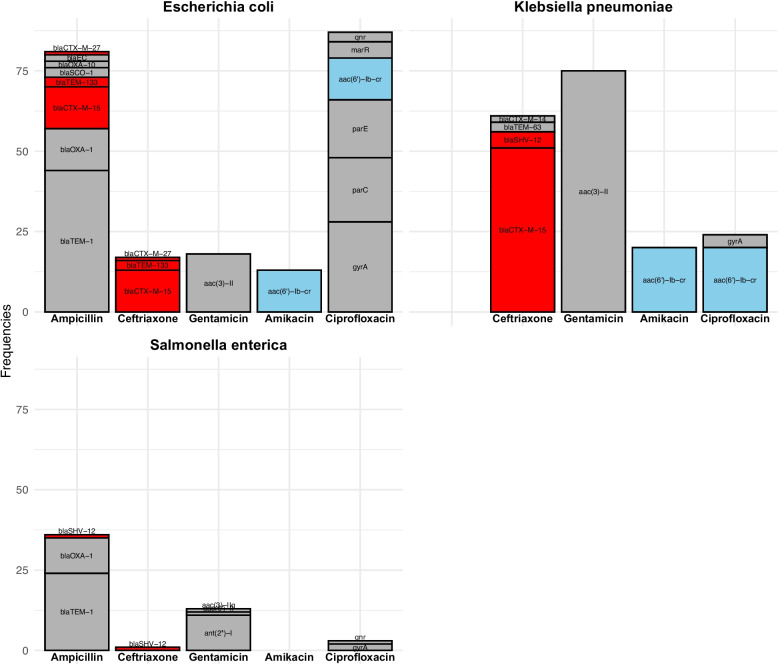
Frequencies of resistance genes (absolute numbers) across 111 *K. pneumoniae*, 59 *E. coli*, and 46 *S. enterica* isolates grouped by which antibiotics they confer resistance to (X-axis). Ampicillin excluded for *K. pneumoniae*. Mutations in quinolone resistance-determining regions grouped in gyrA, parC, parE, and marR. ESBL genes are marked red; aac(6′)-Ib-cr blue

**Fig. 4:**
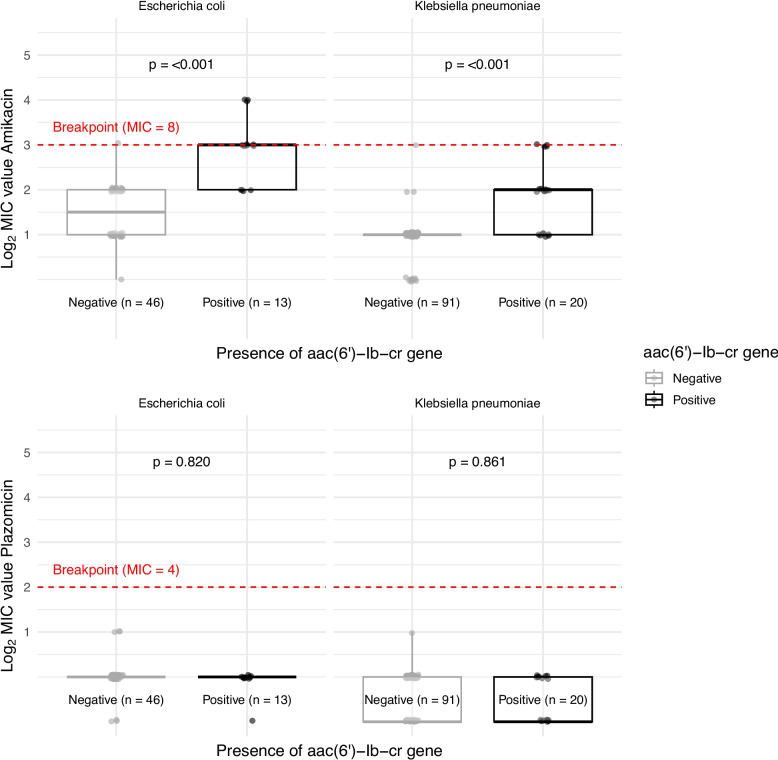
Log_2_-transformed MIC values for amikacin and plazomicin in isolates positive or negative for the aac(6′)-Ib-cr gene. Differences in MIC values assessed by Wilcoxon test. MIC, minimum inhibitory concentration

Figure 3 shows antimicrobial resistance genes identified by WGS in *K. pneumoniae*, *E. coli, *and* Salmonella* isolates. Narrow-spectrum β-lactamases were present in all 111 *K. pneumoniae* isolates (including chromosomal *bla*_SHV_ enzymes), in 53 of 59 *E. coli* isolates, and in 24 of 46 *Salmonella* isolates. The most common resistance genes in this group were *bla*_TEM-1_ and *bla*_OXA-1_, with many isolates carrying multiple genes. Among the detected ESBL genes, *bla*_CTX-M-15_ was identified in 51 *K. pneumoniae* and 13 *E. coli* isolates, *bla*_SHV-12_ in 5 *K. pneumoniae* and 1 *Salmonella* Newport isolate, *bla*_TEM-63_ in 3 *K. pneumoniae* isolates, and *bla*_TEM-133_ in 3 *E. coli* isolates. The aac(3)-II gene was found in 75 *K. pneumoniae*, 18 *E. coli,* and one *S.* Newport isolate, with all but one *Klebsiella* isolate being resistant to gentamicin. The ant(2″)-I gene was found in 11 *Salmonella* isolates, all of which were *S.* Typhimurium sequence type 313 and resistant to gentamicin. The aac(6′)-Ib-cr gene was present in 20 *K. pneumoniae* and 13 *E. coli* isolates. Of these 33 isolates, only three *E. coli* isolates showed phenotypic resistance to amikacin, however MIC values were significantly higher in isolates harbouring the aac(6′)-Ib-cr gene with median MIC 4 (interquartile range (IQR) 4) compared to median MIC 2 (IQR 0) in gene-negative isolates with a Wilcoxon p value < 0.001 (Fig. 4). The presence of the aac(6′)-Ib-cr gene did not influence the MIC values for plazomicin (Fig. 4).

The trend in resistance gene prevalences between our two studies is shown in Supplementary Fig. 3. The prevalence of narrow-spectrum β-lactamases increased from 85 to 89% between Study 1 and Study 2 across all sequenced *K. pneumoniae*, *E. coli*, and *Salmonella* isolates. The prevalence of ESBL genes increased from 14 to 64%, the aac(3)-II gene from 30 to 60%, the aac(6′)-Ib-cr gene from 5 to 28%, and mutations in the quinolone resistance determining region (QRDR) increased from 10 to 16%.

## Discussion

Clinicians treating children with suspected sepsis in LMICs in sub-Saharan Africa face challenges in selecting appropriate empiric antibiotic treatment due to widespread antimicrobial resistance and the lack of affordable diagnostic tools. Blood cultures are often unavailable or unaffordable, leading to non-targeted treatment. Ineffective treatment due to antimicrobial resistance increases morbidity and mortality [7], and the overuse of antibiotics promotes the emergence of increasingly resistant bacteria. The World Health Organisation (WHO) recommends a penicillin combined with gentamicin as the first choice, and ceftriaxone or cloxacillin combined with amikacin as the second choice for empiric sepsis treatment in neonates and older children [42, 43]. Tanzanian national guidelines recommend gentamicin-ampicillin-cloxacillin as an empiric treatment for sepsis, with cefotaxime or ceftriaxone as a secondary option [44–46]. Amikacin combination therapy is currently being evaluated for neonatal sepsis in the NeoSep1 trial [47].

This study finds a favourable susceptibility profile in blood culture isolates to amikacin-ampicillin and plazomicin-ampicillin compared to exceedingly high resistance rates to currently used regimens with gentamicin-ampicillin and ceftriaxone. Overall resistance to gentamicin-ampicillin was 46%, and resistance to ceftriaxone was 50%, compared to 9% resistance to amikacin-ampicillin, with an absolute risk difference of 37 percentage points and 41 percentage points, respectively. The differences were most marked in *Klebsiella*, which was the predominant genus in our dataset. Compared with resistance rates reported in a systematic review of antimicrobial resistance in children in sub-Saharan Africa (2005–2015), our study found higher resistance to gentamicin (66% vs 49%) and ceftriaxone (57% vs 33–50%) in *Klebsiella *spp., and higher resistance to ceftriaxone in *E. coli* (29% vs 16%), whereas resistance to cloxacillin in *S. aureus* was similar [13]. A meta-analysis of antimicrobial resistance in invasive neonatal bacterial infections in sub-Saharan Africa (2008–2018) reported resistance patterns to ceftriaxone, gentamicin, and amikacin in *Klebsiella pneumoniae* that were consistent with those observed in our study, whereas resistance rates to gentamicin in *E. coli* were reported to be higher than in our study (47% vs 25%) [12]. Cloxacillin resistance in *S. aureus* was also reported to be higher than in neonates in our study (40% vs 27%, Supplementary Table 1) [12]. A study of AMR in *Klebsiella pneumoniae* bloodstream infections in Kenya (2001–2011) reported increasing resistance to gentamicin–ampicillin over the study period and consistently low resistance to amikacin, findings that align with those observed in our study [48].

Among the Gram-negative bacteria, resistance to amikacin was rare, occurring in three *E. coli*, one *Proteus sp.*, and three *Acinetobacter *spp. Meropenem resistance was similarly uncommon and observed only in three *Acinetobacter * spp. and one *Elizabethkingia anophelis* based on disc diffusion testing. Meropenem resistance was not reassessed in this study. Resistance rates to gentamicin-ampicillin, ceftriaxone and ciprofloxacin increased between Study 1 (2001–2002) and Study 2 (2017–2018). Resistance rates to plazomicin and amikacin were similar and did not increase between the two studies. Despite higher resistance rates, mortality in paediatric patients with bloodstream infections was lower in Study 2 (8%) than in Study 1 (35%). Study 1 was conducted exclusively at a tertiary hospital, which could partly explain the difference. Several other factors might contribute to lower case-fatality rate, including improvements in healthcare services, lower prevalence of HIV-induced immunodeficiency, malaria, and malnutrition [49–51]. A meta-analysis supports a trend towards declining case-fatality rates in paediatric sepsis in developing countries in the same time period [52].

The aminoglycosides are effective against Gram-negatives and *S. aureus* in the bloodstream, and ampicillin has complementary coverage with effect against streptococci, enterococci, and anaerobes, making the combination favourable. While broad-spectrum β-lactams exert a substantial ecological impact and strongly promote the emergence of antimicrobial resistance, aminoglycosides are mostly excreted in the urine and exert a lesser selective pressure on the gut microbiota [20–23]. AWaRe (Access Watch Reserve) is a system developed by WHO to classify antibiotics based on their potential to promote resistance [53]. The AWaRe Book provides guidance for how to use antibiotics for common infections, prioritizing Access antibiotics [42]. Gentamicin, ampicillin, and amikacin are all listed in the Access group, while ceftriaxone is listed in the Watch group, and meropenem in the Reserve group. A meta-analysis found colonisation with multidrug-resistant bacteria to be more strongly associated with exposure for Watch and Reserve than Access group antibiotics [19]. With the high rate of ESBL-producing *Enterobacterales* in Tanzania, carbapenems remain the only reliable β-lactams; however, using them as first-line empiric treatment would likely accelerate the emergence of carbapenemase-producing organisms, which are already appearing in the region [54]. While aminoglycoside-based treatment has been a cornerstone in the treatment of serious infections and sepsis for years, its clinical effectiveness is less well documented, with most clinical studies being outdated and involving suboptimal dosing [55, 56]. There is controversy about the effectiveness of treating infections other than those originating in the urinary tract with aminoglycosides in monotherapy [56]. Although most studies report a low risk of aminoglycoside-induced nephrotoxicity and ototoxicity in children, others have documented substantially higher toxicity [57]. Studies on adults indicate a low risk with short courses [58–62].

Ceftriaxone resistance was mainly due to the *bla*_CTX-M-15_ gene, with its prevalence increasing substantially between the two studies. Gentamicin resistance was primarily caused by the aac(3)-II gene in *Klebsiella pneumoniae* and *E. coli*, while the ant(2″)-I gene was most common in *Salmonella* isolates. The aac(6′)-Ib-cr gene, often co-localized with *bla*_CTX-M-15_ in *Enterobacterales*, is the major determinant of amikacin resistance and also acetylates fluoroquinolones, thereby reducing ciprofloxacin susceptibility [24, 63, 64]. Although only three *E. coli* isolates were resistant to amikacin, the aac(6′)-Ib-cr gene was found in an additional 20 *Klebsiella pneumoniae* and 10 *E. coli* isolates without causing phenotypic resistance. The spread of the aac(6′)-Ib-cr gene in *Enterobacterales* without raising the MIC above the susceptibility breakpoint is also noted by others, and might be linked with low-level resistance [65–67]. Heterogeneity within the AAC(6′)-Ib-cr molecule with possible different affinity to amikacin, and the different level of expression and intracellular concentration of AAC(6′)-Ib-cr, might explain this [63, 68]. The unchanged MIC values for plazomicin in the presence of this gene suggest that no additional co-localized resistance mechanisms, such as efflux pumps, are involved. The prevalence of the aac(6′)-Ib-cr gene increased from 5 to 28% between the two studies, and the continuous spread of this gene raises concerns about increasing amikacin resistance in the future. Plazomicin-based treatment could be an option to counteract this threat.

A strength of this study is that participants were recruited in well-designed prospective cohort studies, providing much-needed unbiased data on aetiology and antimicrobial resistance of bloodstream infections in sub-Saharan Africa. The in vitro resistance determination of MICs and the analysis of WGS data offer valuable insights into resistance patterns. It is a limitation that the constituent studies are several years old; however, this enables analysis of changes in AMR over time. Future analysis with more recent data will further strengthen this knowledge base. Comparable approaches are being used to infer the evolutionary dynamics of AMR features over time in specific pathogens [69, 70]. Only one aerobic blood culture bottle was collected per patient, which prevented us from distinguishing contamination from true infection for organisms such as coagulase-negative staphylococci. These were therefore excluded, although some may have represented true infections that could warrant empirical coverage. Another limitation is the difference in study settings. Study 1 was conducted exclusively at a tertiary hospital, where patients are typically more severely ill, and referral patterns may result in a higher prevalence of antimicrobial resistance. In contrast, most patients in Study 2 were recruited at regional hospitals. These differences likely influence the apparent temporal changes in AMR observed between the two studies. Although the true direction and magnitude of this effect cannot be determined with certainty, it is plausible that the observed rise in resistance is an underestimate, and the findings should be interpreted with caution. Importantly, while amikacin–ampicillin showed favourable in vitro susceptibility profiles in this study, these findings should not be interpreted as direct evidence for clinical effectiveness. The study did not include clinical outcome measures, pharmacokinetic data, or toxicity assessments, and aminoglycoside safety profiles may differ substantially in resource-limited settings where monitoring of renal function and drug levels is often restricted*.* Consequently, the clinical applicability of amikacin–ampicillin remains uncertain, and there is a clear need to evaluate clinical efficacy and safety in prospective clinical trials.

## Conclusion

Resistance rates to established first-line empiric treatment for paediatric sepsis in Tanzania were already alarmingly high in 2001 and have continued to rise since. Timely revision of the guidelines is urgently needed. The results indicate that amikacin-ampicillin is a promising option as initial empiric sepsis therapy in this population. However, the findings are based on in vitro susceptibility data only, and we need new well-designed clinical trials to assess the clinical effectiveness and safety of this regimen, as well as its impact on gut microbiome and colonization with multi-drug-resistant bacteria.

## Supplementary Information

Supplementary Material 1.

## Data Availability

The whole-genome sequences of all bacterial isolates analysed in this work are publicly available as BioProject ID PRJNA1405348 on this link: http://www.ncbi.nlm.nih.gov/bioproject/1405348. Other study data, codebook and R code for analysis and visualisation are available at the following repository: https://github.com/trygvetrophomonas/BSI-Tanzania.

## Abbreviations

AMR: Antimicrobial resistance
AWaRe: Access Watch Reserve system, WHO
CLSI: Clinical and laboratory standards institute
ESBL: Extended-spectrum beta-lactamase
EUCAST: European committee on antimicrobial susceptibility testing
LMICs: Low- and middle-income countries
MIC: Minimum inhibitory concentration
PCR: Polymerase chain reaction
QRDR: Quinolone resistance-determining region
WGS: Whole genome sequencing
WHO: World Health Organization
