# Supplementary material for: Antimicrobial resistance in paediatric bloodstream infections in Tanzania: a longitudinal comparison of two cohort studies
Source: BMC Microbiol. 2026 Apr 14;26:499. doi: 10.1186/s12866-026-05024-5 (PMC13202842; doi:10.1186/s12866-026-05024-5)
Supplement: Supplementary file 1 — Supplementary Material 1. [file 12866_2026_5024_MOESM1_ESM.docx]

**Supplementary Table 1**. Resistance rates to different treatment regimens in neonates

|  | Gentamicin + Ampicillin | Gentamicin + Ampicillin + Cloxacillin | Ceftriaxone | Ciprofloxacin | Amikacin + Ampicillin | Plazomicin + Ampicillin | Amikacin + Ceftriaxone |
| --- | --- | --- | --- | --- | --- | --- | --- |
| Gram-negative bacteria | | | | | | | |
| *Klebsiella pneumoniae* (N=80) | 64% (n=51) | 64% (n=51) | 60% (n=48) | 14% (n=11) | 0% (n=0) | 0% (n=0) | 0% (n=0) |
| *Salmonella enterica* (N=3) | 0% (n=0) | 0% (n=0) | 0% (n=0) | 0% (n=0) | 0% (n=0) | 0% (n=0) | 0% (n=0) |
| *Escherichia coli* (N=32) | 25% (n=8) | 25% (n=8) | 31% (n=10) | 28% (n=9) | 3% (n=1) | 0% (n=0) | 3% (n=1) |
| Other Gram negatives* (N=18) | 17% (n=3) | 17% (n=3) | 22% (n=4) | 6% (n=1) | 0% (n=0) | 6% (n=1) | 0% (n=0) |
| *Pseudomonas spp.* (N=3) | 100% (n=3) | 100% (n=3) | 100% (n=3) | 33% (n=1) | 0% (n=0) | 0% (n=0) | 0% (n=0) |
| *Acinetobacter* *spp.* (N=7) | 71% (n=5) | 71% (n=5) | 100% (n=7) | 29% (n=2) | 14% (n=1) | 0% (n=0) | 14% (n=1) |
| Gram-positive bacteria | | | | | | | |
| *Staphylococcus aureus* (N=41) | 27% (n=11) | 22% (n=9) | 27% (n=11) | 22% (n=9) | 0% (n=0) | 0% (n=0) | 0% (n=0) |
| *Enterococcus spp.* (N=27) | 67% (n=18) | 67% (n=18) | 100% (n=27) | 100% (n=27) | 67% (n=18) | 67% (n=18) | 100% (n=27) |
| *Streptococcus spp.* (N=4) | 0% (n=0) | 0% (n=0) | 0% (n=0) | 100% (n=4) | 0% (n=0) | 0% (n=0) | 0% (n=0) |
| Polymicrobial | | | | | | | |
| Polymicrobial (N=8) | 62% (n=5) | 62% (n=5) | 75% (n=6) | 38% (n=3) | 0% (n=0) | 0% (n=0) | 25% (n=2) |
|  | | | | | | | |
| Overall (N=223) | 47% (n=104) | 46% (n=102) | 52% (n=116) | 30% (n=67) | 9% (n=20) | 9% (n=19) | 14% (n=31) |

**Supplementary Table 2**. Resistance rates to different treatment regimens in children >28 days

|  | Gentamicin + Ampicillin | Gentamicin + Ampicillin + Cloxacillin | Ceftriaxone | Ciprofloxacin | Amikacin + Ampicillin | Plazomicin + Ampicillin | Amikacin + Ceftriaxone |
| --- | --- | --- | --- | --- | --- | --- | --- |
| Gram-negative bacteria | | | | | | | |
| *Klebsiella pneumoniae* (N=20) | 75% (n=15) | 75% (n=15) | 45% (n=9) | 25% (n=5) | 0% (n=0) | 0% (n=0) | 0% (n=0) |
| *Salmonella enterica* (N=39) | 33% (n=13) | 33% (n=13) | 3% (n=1) | 8% (n=3) | 0% (n=0) | 0% (n=0) | 0% (n=0) |
| *Escherichia coli* (N=23) | 43% (n=10) | 43% (n=10) | 26% (n=6) | 17% (n=4) | 4% (n=1) | 0% (n=0) | 4% (n=1) |
| Other Gram negatives* (N=12) | 25% (n=3) | 25% (n=3) | 17% (n=2) | 8% (n=1) | 0% (n=0) | 0% (n=0) | 0% (n=0) |
| *Pseudomonas spp.* (N=19) | 100% (n=19) | 100% (n=19) | 100% (n=19) | 0% (n=0) | 0% (n=0) | 0% (n=0) | 0% (n=0) |
| *Acinetobacter* *spp.* (N=15) | 40% (n=6) | 40% (n=6) | 100% (n=15) | 13% (n=2) | 13% (n=2) | 13% (n=2) | 13% (n=2) |
| Gram-positive bacteria | | | | | | | |
| *Staphylococcus aureus* (N=26) | 19% (n=5) | 4% (n=1) | 8% (n=2) | 4% (n=1) | 0% (n=0) | 0% (n=0) | 0% (n=0) |
| *Enterococcus spp.* (N=36) | 39% (n=14) | 39% (n=14) | 100% (n=36) | 100% (n=36) | 39% (n=14) | 39% (n=14) | 100% (n=36) |
| *Streptococcus spp.* (N=2) | 0% (n=0) | 0% (n=0) | 0% (n=0) | 100% (n=2) | 0% (n=0) | 0% (n=0) | 0% (n=0) |
| Polymicrobial | | | | | | | |
| Polymicrobial (N=12) | 75% (n=9) | 75% (n=9) | 67% (n=8) | 42% (n=5) | 25% (n=3) | 25% (n=3) | 33% (n=4) |
|  | | | | | | | |
| Overall (N=204) | 46% (n=94) | 44% (n=90) | 48% (n=98) | 29% (n=59) | 10% (n=20) | 9% (n=19) | 21% (n=43) |

**Supplementary Table 3**. Resistance rates to different treatment regimens - Study 1

|  | Gentamicin + Ampicillin | Gentamicin + Ampicillin + Cloxacillin | Ceftriaxone | Ciprofloxacin | Amikacin + Ampicillin | Plazomicin + Ampicillin | Amikacin + Ceftriaxone |
| --- | --- | --- | --- | --- | --- | --- | --- |
| Gram-negative bacteria | | | | | | | |
| *Klebsiella pneumoniae* (N=40) | 45% (n=18) | 45% (n=18) | 18% (n=7) | 0% (n=0) | 0% (n=0) | 0% (n=0) | 0% (n=0) |
| *Salmonella enterica* (N=33) | 39% (n=13) | 39% (n=13) | 3% (n=1) | 3% (n=1) | 0% (n=0) | 0% (n=0) | 0% (n=0) |
| *Escherichia coli* (N=29) | 34% (n=10) | 34% (n=10) | 21% (n=6) | 10% (n=3) | 3% (n=1) | 0% (n=0) | 3% (n=1) |
| Other Gram negatives* (N=10) | 50% (n=5) | 50% (n=5) | 50% (n=5) | 10% (n=1) | 0% (n=0) | 10% (n=1) | 0% (n=0) |
| *Pseudomonas spp.* (N=11) | 100% (n=11) | 100% (n=11) | 100% (n=11) | 0% (n=0) | 0% (n=0) | 0% (n=0) | 0% (n=0) |
| *Acinetobacter* *spp.* (N=11) | 64% (n=7) | 64% (n=7) | 100% (n=11) | 9% (n=1) | 18% (n=2) | 18% (n=2) | 18% (n=2) |
| Gram-positive  bacteria | | | | | | | |
| *Staphylococcus aureus* (N=26) | 12% (n=3) | 4% (n=1) | 12% (n=3) | 4% (n=1) | 0% (n=0) | 0% (n=0) | 0% (n=0) |
| *Enterococcus spp.* (N=33) | 45% (n=15) | 45% (n=15) | 100% (n=33) | 100% (n=33) | 45% (n=15) | 45% (n=15) | 100% (n=33) |
| *Streptococcus spp.* (N=2) | 0% (n=0) | 0% (n=0) | 0% (n=0) | 100% (n=2) | 0% (n=0) | 0% (n=0) | 0% (n=0) |
| Polymicrobial | | | | | | | |
| Polymicrobial (N=16) | 69% (n=11) | 69% (n=11) | 62% (n=10) | 38% (n=6) | 12% (n=2) | 12% (n=2) | 25% (n=4) |
|  | | | | | | | |
| Overall (N=211) | 44% (n=93) | 43% (n=91) | 41% (n=87) | 23% (n=48) | 9% (n=20) | 9% (n=20) | 19% (n=40) |

**Supplementary Table 4**. Resistance rates to different treatment regimens - Study 2

|  | Gentamicin + Ampicillin | Gentamicin + Ampicillin + Cloxacillin | Ceftriaxone | Ciprofloxacin | Amikacin + Ampicillin | Plazomicin + Ampicillin | Amikacin + Ceftriaxone |
| --- | --- | --- | --- | --- | --- | --- | --- |
| Gram-negative  bacteria | | | | | | | |
| *Klebsiella pneumoniae* (N=60) | 80% (n=48) | 80% (n=48) | 83% (n=50) | 27% (n=16) | 0% (n=0) | 0% (n=0) | 0% (n=0) |
| *Salmonella enterica* (N=9) | 0% (n=0) | 0% (n=0) | 0% (n=0) | 22% (n=2) | 0% (n=0) | 0% (n=0) | 0% (n=0) |
| *Escherichia coli* (N=26) | 31% (n=8) | 31% (n=8) | 38% (n=10) | 38% (n=10) | 4% (n=1) | 0% (n=0) | 4% (n=1) |
| Other Gram negatives* (N=20) | 5% (n=1) | 5% (n=1) | 5% (n=1) | 5% (n=1) | 0% (n=0) | 0% (n=0) | 0% (n=0) |
| *Pseudomonas spp.* (N=11) | 100% (n=11) | 100% (n=11) | 100% (n=11) | 9% (n=1) | 0% (n=0) | 0% (n=0) | 0% (n=0) |
| *Acinetobacter* *spp.* (N=11) | 36% (n=4) | 36% (n=4) | 100% (n=11) | 27% (n=3) | 9% (n=1) | 0% (n=0) | 9% (n=1) |
| Gram-positive  bacteria | | | | | | | |
| *Staphylococcus aureus* (N=41) | 32% (n=13) | 22% (n=9) | 24% (n=10) | 22% (n=9) | 0% (n=0) | 0% (n=0) | 0% (n=0) |
| *Enterococcus spp.* (N=30) | 57% (n=17) | 57% (n=17) | 100% (n=30) | 100% (n=30) | 57% (n=17) | 57% (n=17) | 100% (n=30) |
| *Streptococcus spp.* (N=4) | 0% (n=0) | 0% (n=0) | 0% (n=0) | 100% (n=4) | 0% (n=0) | 0% (n=0) | 0% (n=0) |
| Polymicrobial | | | | | | | |
| Polymicrobial (N=4) | 75% (n=3) | 75% (n=3) | 100% (n=4) | 50% (n=2) | 25% (n=1) | 25% (n=1) | 50% (n=2) |
|  | | | | | | | |
| Overall (N=216) | 49% (n=105) | 47% (n=101) | 59% (n=127) | 36% (n=78) | 9% (n=20) | 8% (n=18) | 16% (n=34) |

**Supplementary Figure 1**. MIC-distributions Amikacin.

**Supplementary Figure 2a**. Absolute risk differences for resistance to Amikacin- Ampicillin compared to Gentamicin-Ampicillin for neonates

**Supplementary Figure 2b**. Absolute risk differences for resistance to Amikacin- Ampicillin compared to Ceftriaxone for neonates

**Supplementary Figure 2c**. Absolute risk differences for resistance to Amikacin- Ampicillin compared to Gentamicin- Ampicillin for children >28 days

**Supplementary Figure 2d**. Absolute risk differences for resistance to Amikacin-Ampicillin compared to Ceftriaxone for children <28 days


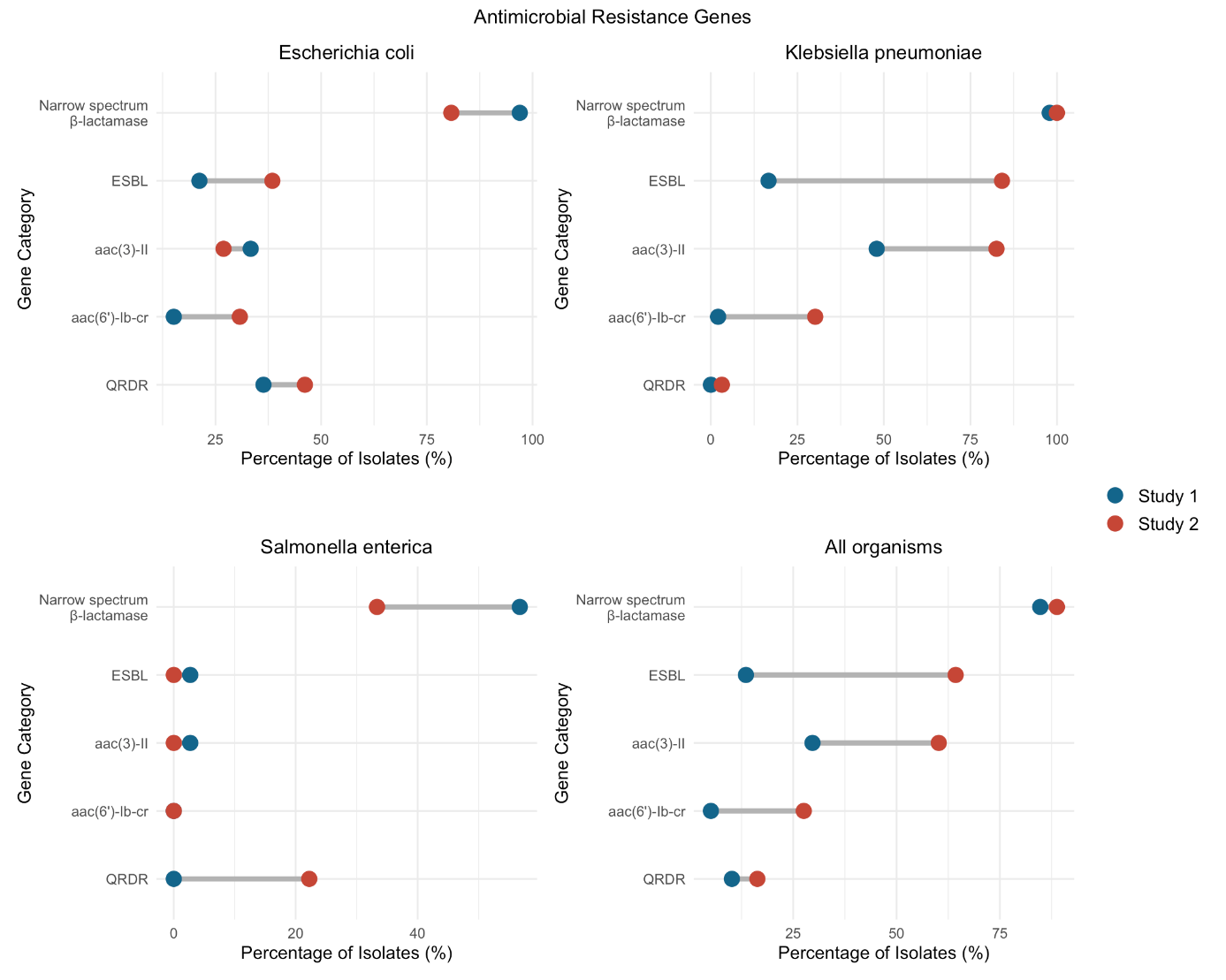


**Supplementary Figure 3.** Change in prevalence of important resistance genes and resistance gene groups between Study 1 and Study 2 across *E. coli*, *K. pneumoniae*, and *S. enterica* isolates.
